# Barriers to the use of reminder/recall interventions for immunizations: a systematic review

**DOI:** 10.1186/1472-6947-12-145

**Published:** 2012-12-17

**Authors:** Jennifer A Pereira, Susan Quach, Christine L Heidebrecht, Sherman D Quan, Faron Kolbe, Michael Finkelstein, Jeffrey C Kwong

**Affiliations:** 1 Surveillance and Epidemiology, Public Health Ontario, 480 University Ave., Suite 300, Toronto, ON, M5G 1V2, Canada; 2 University Health Network, Toronto, Canada; 3 Toronto Public Health, Toronto, Canada; 4 Dalla Lana School of Public Health, University of Toronto, Toronto, Canada; 5 Department of Family and Community Medicine, University of Toronto, Toronto, Canada; 6 Institute for Clinical Evaluative Sciences, Toronto, Canada

**Keywords:** Reminder, Recall, Immunization, Systematic review, Barriers

## Abstract

**Background:**

Although many studies have demonstrated the benefits of reminder/recall (RR) measures to address patient under-immunization and improve immunization coverage, they are not widely implemented by healthcare providers. We identified providers’ perceived barriers to their use from existing literature.

**Methods:**

We conducted a systematic review of relevant articles published in English between January 1990 and July 2011 that examined the perceptions of healthcare providers regarding barriers to tracking patient immunization history and implementing RR interventions. We searched MEDLINE, PubMed, EMBASE, Cumulative Index to Nursing and Allied Health Literature, Academic Search Premier, and PsychINFO. Additional strategies included hand-searching the references of pertinent articles and related reviews, and searching keywords in Google Scholar and Google.

**Results:**

Ten articles were included; all described populations in the United States, and examined perceptions of family physicians, pediatricians, and other immunization staff. All articles were of moderate-high methodological quality; the majority (n=7) employed survey methodology. The most frequently described barriers involved the perceived human and financial resources associated with implementing an RR intervention, as well as low confidence in the accuracy of patient immunization records, given the lack of data sharing between multiple immunization providers. Changes to staff workflow, lack of appropriate electronic patient-tracking functionalities, and uncertainty regarding the success of RR interventions were also viewed as barriers to their adoption.

**Conclusions:**

Although transitioning to electronic immunization records and registries should facilitate the implementation of RR interventions, numerous perceived barriers must still be overcome before the full benefits of these methods can be realized.

## Background

Although immunization is the most effective defense against vaccine preventable diseases, vaccine coverage is suboptimal in many populations [1-3]. Barriers to immunization have been well-studied and include anti-vaccination sentiments, difficulty accessing a healthcare provider (HCP) to provide immunization, public perception of a lack of endorsement by physicians, and, for non-publicly funded vaccines, cost. However, one of the most commonly cited reasons for low coverage is patients or their caregivers being unaware that one or more vaccines are due or overdue [4-8].

To address this prevalent issue, standalone and multi-faceted interventions have been developed to improve vaccine coverage, both to remind individuals of upcoming immunizations and to recall those for whom immunizations are overdue. Such interventions have been termed "reminder/recall" (RR) measures, and methods include automated or personal telephone calls, postcards, letters, and text messages to patients [9-13]. RR interventions can also be directed at immunization providers using processes such as reminders attached by nurses or receptionists to patient charts and, with the growth of electronic medical records (EMRs) and registries, through computerized alerts [14,15]. Regardless of whom the RR systems are targeting, they have been demonstrated to increase vaccine coverage by 5-20% [16-18].

The benefits of RR interventions may also extend to data quality, since such measures may prompt patients to update other personal information, thereby leading to improved accuracy and completeness of the entire patient record. Furthermore, patients who are under-immunized often do not participate in other recommended preventive care activities; immunization-based RR can improve this by providing opportunities for HCP-patient interactions [19].

RR interventions start with the identification of patients who are due or overdue for immunization, involving either comparisons of electronic records with a system-embedded schedule or manual chart reviews where electronic records are not available. Although electronic systems should facilitate the efficient identification of under-immunized patients and those soon to be due [20], only 15-25% of physicians identify patients who are not up-of-date with vaccinations and implement an RR intervention [21-23]. It is important to understand why, despite the known effectiveness of RR measures and the increasing availability of this functionality associated with growing EMR use, these practices are not being adopted by immunization providers. We therefore conducted a systematic review to explore what providers perceive as barriers to the utilization of RR interventions.

## Methods

Prior to conducting the review, our study team defined its parameters:

i. Study scope: Our search focused on summarizing the barriers identified by immunization providers (including physicians, nurses, and pharmacists) toward implementing either provider-directed RR interventions (measures to deliver reminders to immunization providers) or patient-directed interventions (client interventions initiated by immunization providers). Methodologies to elicit provider views may include surveys, focus groups, or interviews.

ii. Search process: The systematic search retrieved articles using criteria developed based on input from the study team, keywords used in related previous reviews, and consultation with an information specialist.

iii. Quality assessment: Each paper was evaluated using an amalgamation of previous tools to assess the rigour of methodology and the quality of reporting.

iv. Barrier summary: We extracted the reported barriers and grouped them where possible, based on any noted commonalities.

### Literature search and study selection

We searched MEDLINE, PubMed, EMBASE, Cumulative Index to Nursing and Allied Health Literature, Academic Search Premier, and PsychINFO for articles published in January 1990 through July 2011 on barriers to the use of immunization RR interventions, using various combinations of Medical Subject Headings terms (e.g., immunization, immunization programs, vaccination, appointment and schedules, reminder systems, parental notification) and keywords (e.g., recall, reminder, barrier, attitude, behavior, adopt). Search criteria were developed based on consultation with a library information specialist, as well as reviewing the keywords used in previous reviews of RR interventions. Full search criteria are described in Table 1. Articles were considered eligible for evaluation if they were in English, contained original data, and described studies using quantitative and/or qualitative methodologies to identify the barriers perceived by immunization staff towards implementing RR interventions for childhood and/or adult immunizations. The perceived barriers could be towards any type of RR intervention for immunization directed at patients/their caregivers or healthcare providers. We excluded reviews, editorials, commentaries, and practice guidelines, as well as conference abstracts and other non-full text publications.

**Table 1:** Literature search terms by database

**Search Engine**^**^**^	**Search Terms (MeSH and Keywords)**^**~**^	**Number of citations**
MEDLINE	(exp *immunization* OR exp *immunization program* OR *vaccin$* OR *immun$)*	631
	AND	
	(exp *appointments and schedules* OR exp *reminder systems* OR exp *parental notification* OR *guideline adherence* OR *alert$* OR *reminder$* OR *notification$* OR *recall$* OR *appointment$)*	
	AND	
	(exp *attitude* OR *barrier$* OR *challeng$* OR *obstacle$* OR *adopt$* OR *attitude$* OR *behaviour$)*	
Cumulative Index to Nursing and Allied Health Literature (CINAHL)	(exp *immunization* OR exp *immunization programs* OR or vaccin*)	233
	AND	
	(exp *appointment and scheduling information systems* OR exp *appointments and schedules* OR exp *reminder systems* or exp *parental notification* OR exp *guideline adherence* OR alert* OR reminder* OR notification* OR recall* OR appointment*)	
	OR	
	(exp barrier* OR challeng* OR obstacle* OR adopt* OR attitude* OR behaviour*)	
Academic Search Premier (ASP)	(exp *immunization* OR exp *immunization programs* OR or vaccin*)	178
	AND	
	(exp *appointment and scheduling information systems* OR exp *appointments and schedules* OR exp *reminder systems* or exp *parental notification* OR exp *guideline adherence* OR alert* OR reminder* OR notification* OR recall* OR appointment*)	
	OR	
	(exp barrier* OR challeng* OR obstacle* OR adopt* OR attitude* OR behaviour*)	
PsychINFO	(exp *immunization* OR exp *immunization programs* OR or vaccin*)	18
	AND	
	(exp *appointment and scheduling information systems* OR exp *appointments and schedules* OR exp *reminder systems* or exp *parental notification* OR exp *guideline adherence* OR alert* OR reminder* OR notification* OR recall* OR appointment*)	
	OR	
	(exp barrier* OR challeng* OR obstacle* OR adopt* OR attitude* OR behaviour*)	
PubMed	(exp *immunization* OR exp *immunization programs* OR exp *vaccination* OR immuni* OR vaccin*)	877
	AND	
	(exp *appointments and schedules* OR exp *reminder systems* OR exp *parental notification* OR exp *guideline adherence* OR alert* OR reminder* OR notification* OR recall* OR appointment*)	
	AND	
	(barrier* OR challeng* OR obstacle* OR adopt* OR exp *attitude* OR *attitude* OR *behaviour*)	
EMBASE	(exp *immunization/*OR *immunization program* OR exp *vaccination*/OR *vaccin$* OR *immuni$*)	1291
	AND	
	(exp *reminder system/*OR exp *parental notification/*OR guideline adher* OR appointment* OR schedul* OR remind* OR *alert$* OR *notif$* OR *recall$* OR *information technology* OR exp *information system/*)	
	AND	
	(barrier* OR challeng* OR obstacle* OR adopt* OR attitud* OR *behavio#r* OR utili#ation* OR utili*)	
***TOTAL:***		**3227**

This table describes the databases and search terms used in the systematic review, to identify articles on barriers to the use of reminder/recall interventions.

^^^Searches restricted to articles published between January 1990 and July 2011, and in English.

^~^exp = expanded search term.

We entered the titles of relevant articles into the PubMed “related articles” feature and also hand-searched the bibliographies of review papers, relevant articles, and systematic reviews from the Cochrane Database of Reviews of Effectiveness to identify additional pertinent papers. We used Google Scholar and Google to search the grey literature for government reports and other documents.

### Data abstraction/outcomes

**Figure 1:**
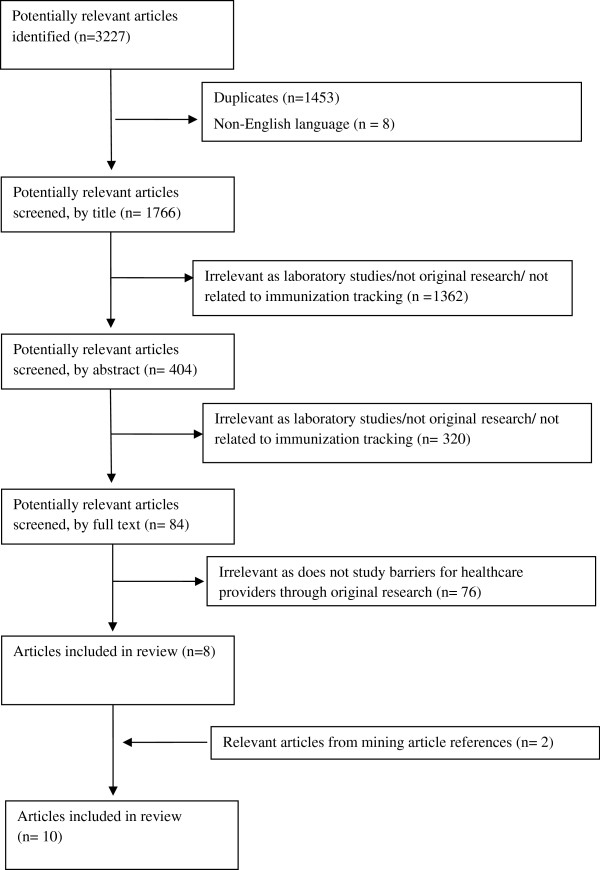
This figure depicts the filter process for articles identified by our search strategies.

After duplicate articles were removed, all titles and abstracts were reviewed for relevance (Figure 1). Those articles with abstracts pertaining to our main objectives were then reviewed in full-text. JP conducted the primary review; a second reviewer (SQ) was consulted to verify the final list of articles, as well as to confer on all articles for which there was uncertainty as to whether inclusion criteria were met. A consensus on each of these articles was reached.

### Methodological quality assessment

JP and SQ independently assessed the methodological rigour of all eligible articles based on a modification of tools designed to assess qualitative and quantitative studies (the Critical Appraisal Skills Programme (CASP) appraisal tool and Centre for Evidence-Based Management Survey Scoring System, respectively) [24,25]. Articles were evaluated and classified as high/moderate/low quality based on the following 14 elements: clear research aims; appropriate research design; recruitment strategy; representativeness of population; avoidance of selection bias; data collection; sample size considerations; sufficient response rate; validity of measurements; ethical considerations; rigour of data analysis; significance testing; clarity of findings; and applicability of research. We scored one point for each element, for a maximum of 14; articles deemed to be of high methodological quality had ≥ 12 points, moderate = 8 – 11 points, and low = < 8 points. Rating disagreements were infrequent and were resolved through discussion.

A structured review form was then used by both reviewers to abstract data including country, study population, study methodology, and RR intervention(s) studied. The reviewers also independently abstracted any immunization providers’ perceived barriers towards the use of RR measures identified in each paper, and conferred to ensure consistency and completeness. Together, the reviewers then stratified these barriers where appropriate, based on commonalities.

## Results

The structured database searches yielded 1,774 titles. Eight articles were identified as meeting the inclusion criteria. Based on a review of the reference lists of pertinent papers, two additional articles were identified, for a total of 10.

### Descriptive summary

Five of the 10 articles employed surveys [26-30], one used semi-structured interviews [31], two involved a series of focus groups [32,33], and two described a mixed-methodology design (Table 2) [23,34]. All described studies in American populations.

**Table 2:** Summary of eligible articles

**Author, year**	**Methods**	**Participants; setting**	**Intervention/System**	**Barriers**
Birmingham 2011 [32]	Focus group	21 pediatricians/nurse practitioners; New York, US	Computerized clinical reminders (CCRs) for influenza/electronic health records (EHR)	· Too much pop-up information makes it easy to ignore all alerts
				· Mixed confidence in reliability and accuracy of EHR alerts
				· Strongly opposed to alerts that interrupted workflow or forced an action before continuing documentation in a note
				· Concern that alerts will strain nursing staff
Clark 2006 [26]	Mail survey	756/1235 family physicians; 15 states in the US	Patient immunization history tracking for RR interventions/state immunization registry	· Too much cost/staff time
				· Insufficient technology assistance
Dombkowski 2007 [28]	Mail survey	389/600 pediatricians and family physicians; Michigan, US	Patient immunization history tracking for RR interventions/state immunization registry	· Accuracy of Medicaid data used to identify children with asthma and the potential restriction of the registry’s high-risk indicator to only Medicaid patients
				· Consistent access to the registry
				· Overall accuracy and completeness of registry data
				· Staff not accustomed to using registry to check patients’ immunization status
Deutchman 2000 [27]	Mail survey	158/250 family physicians with pediatric patients; rural Colorado, US	Patient immunization history tracking for RR interventions/no specific system	· Integration of new system into current computerized functions
				· Patient confidentiality
				· Costs, staff time associated with using the system to track patients
Fung 2004 [30]	Survey	261/1304 clinical staff or informatics experts from 142 Veterans Health Administration (VHA) facilities; US	CCRs including for immunizations/EHR	· Perceived utility of CCRs, training and personnel support for computer use, EHR functionalities and performance data feedback to providers at each facility
Humiston 2009 [33]	Focus groups	24 family physicians and nurses; New York, US	Patient immunization history tracking for RR interventions/no specific system	· Difficulties in identifying which adolescents were vaccinated, especially due to frequent moves
				· Neither EMR nor state registries are helpful given poor communication between school and primary care offices
Saville 2011 [31]	Semi-structured interviews	24 pediatricians, nurses and practice administrators from 11 practices; Colorado, US	Patient immunization history tracking for RR interventions/state immunization information system	· Difficulties overcoming the obstacle of inaccurate contact information
				· Perceptions of low compliance with recall notices for certain risk groups
				· Perceived conflicts in the immunization algorithms between registry and the practice.
				· Lack of dedicated time and personnel for recall activities
				· Inaccuracies both with patient contact information and immunization data in system;
				patient contact information was not routinely updated in system, only in EHR
				· Unmet expectations for responses to recall efforts can lead to method discontinuation
				· Extra time required to crosscheck recall with appointment schedules to ensure under-immunized patients have not already planned physician visit
Tierney 2003 [23]	Semi-structured interviews and surveys	18 clinician-administrators representing adopters and non-adopters; 912 (76%) pediatricians and public health staff completed surveys; US	Patient immunization history tracking for RR interventions/no specific system	· Both adopters and non-adopters of reminder or recall messages identified time and money as the most important barriers to implementing these methods.
				· Not having a simple way of identifying children at a specific age, review records or begin an initiative
				· Lack of knowledge about how to get started and limited computer skills were named as barriers by only 10% to 18% of respondents in any subgroup
Wallace 2004 [34]	Semi-structured interviews, questionnaires, group discussions	Clinicians at 23 Spinal Cord Injury (SCI) centers in the VHA; US	CCRs for influenza/EHR	· Lack of coordination between EHR and vaccination data so cannot be sure patient has not been vaccinated elsewhere unless extra work is done
				· Different forms (and locations) for inpatients and outpatients is frustrating for clinicians
				· Lack of training can result in inadequate information that is not useful
				· Lack of access for all immunization staff
Yarnall 1998 [29]	Survey	Physicians caring for a sample of 1314 study patients in a large community health centre*; North Carolina, US	CCRs including those for immunizations/computerized health maintenance system	· Lack of time
				· Additional workload as staff still need to use and complete paper maintenance forms

This table summarizes key details of the 10 articles included in the systematic review.

*Number of physicians not reported.

Four articles examined the perceptions of either pediatricians solely or in addition to other HCPs [23,28,31,32] while the remaining articles focused on family physicians and other staff (nurse practitioners, health informatics experts, other clinicians, etc.) [26,27,29,30,33,34]. Six studies identified barriers to the use of RR interventions associated with specific immunization registries and information systems [25,28-31,34].

### Methodological quality

All articles were found to be of moderate (n=6) to high (n=4) methodological quality; there was 100% agreement between the two reviewers on this rating. All articles reported research aims, employed appropriate study design as well as recruitment strategies, utilized a suitable research design, provided sufficient data analysis, and described results with good applicability. While the majority of the other methodological elements examined were also well reported across the studies, only five studies described a satisfactory response rate for their quantitative or qualitative study.

Given the strong methodological quality of the 10 articles, we felt confident in including all in this review.

### Barriers

Although HCPs acknowledged the improvement in immunization coverage associated with RR interventions, they identified several concerns with implementing such measures in their practice settings. These perceived barriers have been grouped into broad themes below.

#### Resources

Financial and human resource constraints were consistently identified as barriers to RR implementation across the majority of articles. Tracking immunization histories of patients was considered very time-consuming, requiring concerted staff time and cost to complete. This was particularly true for those without electronic systems, thereby necessitating manual chart audits to identify those due or overdue for vaccinations [23,26-28,31,32].

For computerized systems, the need for technical assistance, such as support for programming EMRs/registries with immunization algorithms to identify those eligible or overdue for certain vaccines, was also described as an added challenge to the implementation of RR measures [26,27]. For such systems, human resource barriers also included the training needs of staff: respondents described a lack of knowledge regarding how to implement an RR intervention, and uncertainty regarding optimal designs, particularly for staff with limited computer skills [23]. In one article, respondents expressed concern that using an RR measure could lead to the perception of unprofessionalism, should reminders be sent to patients who already have an immunization appointment scheduled for the near future [31]. As a safeguard against this, a cross-check of the appointment schedules for under-immunized patients was added to the workflow prior to issuing reminders, which also added extra time. An additional identified barrier was the increased work load for already busy staff that may result from the appointments of patients identified through the RR process [32].

While financial costs were frequently identified as a barrier to RR implementation in the reviewed articles, they were only broadly discussed; costs related to increased human resources (administrative staff to implement tracking activities, immunization staff to carry out vaccinations, and technical staff to support the interventions) as well as upgraded system functionality, where the intervention was integrated into an electronic system, were described [23,27,31].

#### Data quality

One of the most frequently mentioned barriers to using an RR intervention was the perception of a lack of reliable vaccination data on which to base reminders and recalls [28,31,32]. Identified reasons that a single provider would not have comprehensive immunization records included patients having recently moved, having no regular primary care physician and consequently seeing multiple HCPs, or seeking immunization from a different source such as a public health setting or a school-based clinic. The lack of integrated health systems to facilitate data sharing between multiple providers has resulted in concerns; even if an RR intervention was implemented, it may yield poor results, given that those who were recorded as being under-immunized at their "medical home" may have simply sought the vaccination from a separate provider [28,32].

This perception of poor data quality also extended to contact information, resulting in the concern that the RR intervention would not reach the intended patient [31]. This was also an issue where immunization information was recorded more than once (i.e., in charts as well as a registry); when neither are consistently updated, it is challenging to identify the best source of accurate patient information [31,33].

#### Workflow changes

Workflow barriers were identified as minor concerns associated with the implementation of RR, and included instances where the initiation of an RR activity involved new staff responsibilities, such as checking a state registry for patient immunization status or verifying information [28].

A workflow-related barrier to the use of computerized clinical alerts for immunization is the perception that pop-ups may be disruptive to the patient visit. Since the HCP may already have multiple health issues to address with the patient, the potential volume of such alerts may appear overwhelming, resulting in all messages being ignored [32].

#### System-based issues

Two articles described barriers to RR intervention implementation within specific systems and registries [31,34]; these concerns likely apply to other electronic clinical systems. There was a perception that a single system may not reflect the appropriate immunization algorithms for all of its users, thereby rendering RR interventions ineffective [31].

Complicated systems such as those with different record formats for inpatients and outpatients, or certain high-risk groups, were also perceived to increase the time to implement a reminder alert comprehensively across all eligible patients [34].

Privacy issues associated with the system in use were described in one article: one-third of survey respondents expressed concerns regarding whether using tracking systems to identify patients due or overdue for immunizations and implementing an RR intervention may result in breaches of patient confidentiality [27].

#### Expectations

Varying expectations of the utility of RR interventions were identified by two articles as barriers to its adoption [30,31]. In one study that examined RR specifically for adolescent vaccination, users perceived that caregivers may not be receptive to interventions, perhaps viewing immunizations as less urgent for this age group, compared to younger cohorts [31].

The same article mentioned the belief that for other populations, an RR intervention *should* result in vastly improved coverage; if substantial benefits were not realized after initial use, unmet high expectations were identified as posing a potential barrier to the sustainability of the method [31].

Expectations about healthcare responsibilities also appear to affect willingness to adopt an RR intervention. In a study examining perceptions of RR adopters versus non-adopters, the latter group was less likely to consider all aspects of immunization delivery to be the responsibility of the healthcare system as a whole. There is no impetus to initiate RR interventions when HCPs believe that it is someone else’s duty to remember when a patient’s immunization is due [23].

## Discussion

Although monitoring of patient immunization records continues to expand as HCPs transition to electronic documentation, our review has demonstrated that there are many barriers to the implementation of RR interventions. While several of these issues relate to the perceived workflow changes associated with RR measures, most result from the current limitations surrounding immunization record-keeping.

The human resource requirements expected for RR interventions were identified as a significant barrier to their use in several of the articles reviewed; the perception that additional staff time is required to properly implement such measures is likely intensified by uncertainty over who should be implementing RR features, if anyone. Some HCPs may feel that parents/guardians respond best to notes sent from their child’s physician, others may perceive that measures to ensure appropriate immunization levels are best handled by public health, while still others view remembering immunizations are due as the responsibility of the patient or their caregiver. Efficient RR implementation requires coordination between physicians and local public health agencies to ensure that every individual is accounted for, including those without a primary care physician. Those HCPs with the view that an individual’s health is a shared responsibility across the health system rather than by a single provider seemed to have better acceptance of RR interventions, and perhaps a greater willingness to allocate staff time for their conduct [23].

Cost was also identified as a significant barrier to implementing RR methods. There are a range of interventions available including automated telephone and letter reminders to patients, based on manual chart audits or computerized systems programmed to determine the dates that immunizations are due and generate reminder messages accordingly. Previous studies have compared these methods by outcomes including financial resources required, finding that automated dialing is typically more cost-effective. However, this may depend on the start-up costs needed (for example, in the case of automated calls, the purchase of dialer equipment) and may vary based on target population [35-38]. Certain tools such as computerized reminders to alert the HCP that their patient is due for a vaccination may be a built-in functionality of the EMR system or immunization information system used, and therefore could potentially represent a cost-saving measure.

Optimal use of RR interventions begins with the accurate tracking of patient immunization information; where an electronic system is being used, identifying those individuals eligible for vaccination requires access to an electronic immunization schedule which has been integrated into that system. However, even where a single national vaccine schedule is recommended, implementation may vary locally [39]. This issue is exacerbated in countries where every region is responsible for developing their own schedule and the variations between regions can be considerable; when EMR or other electronic systems are available nationally or are in use in more than one region, RR programming becomes complex. Additionally, if a practice adheres to even slightly different immunization schedules and age eligibilities than those specified in regional recommendations (and incorporated into the system in use), it will be challenging to quickly identify the patients that the practice defines as due or overdue for one or more vaccines. Improved communication between system developers, healthcare providers, and immunization program managers is vital to ensure that users have access to the appropriate schedules in their systems. The use of EMRs or immunization information systems with a region-specific immunization schedule is a key first step in initiation of RR activities if they are to be the responsibility of clinicians.

The studies we reviewed suggest that HCPs lack confidence in the accuracy and completeness of their patient immunization records. Immunization records can become fragmented between local public health departments (for school and non-school based immunizations), primary care physicians, and other HCPs who administer vaccines. Therefore the completeness of any one source of immunization information becomes suspect, limiting the value of any RR intervention based on these data. However, checking multiple places for a patient’s immunization documentation is impractical. A single regional immunization registry that is updated each time a vaccine is administered would be very beneficial. Based on the results of a systematic review, in 2010, the Task Force on Community Preventive Services at the United States Centers for Disease Control and Prevention recommended the use of immunization information systems as a means to increase vaccination rates, through numerous functionalities including RR interventions directed at clients and providers [40]. Using an immunization registry as the source of "truth" would ensure that RR notices are based on complete, up-to-date information from all relevant sources [31,32]. Additionally, since RR interventions will typically be initiated by the primary care provider, the transfer of information from registry to provider could facilitate the process. The benefits of a single source of vaccination information may extend to client safety; a recent study found that children with more than one immunization provider have a higher rate of over-immunization than those with one provider only [41].

Several limitations should be considered when interpreting the results of this review. It is possible that our search strategies may not have identified every pertinent article, particularly those only available in grey literature. Our restriction to English-language−only articles also may have excluded some relevant studies. Additionally, as all articles included in the final review were based on American populations, it is possible that our results may not extend to countries that differ significantly from the U.S. in terms of health care and immunization delivery. We were unable to identify a single tool that was appropriate for assessing quality of both quantitative and qualitative studies; as the one we developed and used for this review is an amalgamation of published resources, we are confident in its comprehensiveness, but acknowledge that it is not yet validated. Finally, we were unable to include a synthesis of results beyond a descriptive summary given the low yield of relevant articles combined with the nature of our research question.

## Conclusions

Despite many routine childhood and adult vaccines being publicly funded for various risk groups in some regions, vaccine coverage is still sub-optimal [1-3]. Although RR interventions have been shown to be an effective means of improving coverage, our review has summarized several reasons that these measures have not been adopted in vaccination settings. Improved training and knowledge about RR interventions will help immunization staff address some of these issues. However, others require changes in data infrastructure, and are more challenging to overcome. Current immunization data collection processes are mainly piecemeal, eroding provider confidence in any one data repository being complete and accurate; to facilitate successful implementation of tracking functionalities, a single trusted source of up-to-date immunization data is required. Until such time that all providers are mandated to update their regional immunization registry for each administered vaccine, improved communication is required among those involved in immunization delivery, including public and private healthcare providers as well as registry and EMR developers, to ensure the comprehensiveness of data collected, system utility, and the incorporation of immunization schedule algorithms that correspond to prospective users’ jurisdictions. The use of RR interventions will be optimized and the benefits best realized when such modifications are made to address current challenges.

## Competing interests

There are no competing interests to report.

## Authors’ contributions

All authors (JP, SQ, CH, SDQ, FK, MF and JCK) participated in the conception and design of this review. JP and SQ conducted the review. All authors were involved in either drafting the manuscript or providing revisions. All authors read and approved the final manuscript.

## Authors’ information

PCIRN Vaccine Coverage Theme Group members are: Julie Bettinger, Nicole Boulianne, Stephanie Brien, David Buckeridge, Larry Chambers, Natasha Crowcroft, Lois Crowe, Shelley Deeks, Michael Finkelstein, Maryse Guay, Jemila Hamid, Christine Heidebrecht, Faron Kolbe, Jeff Kwong, Allison McGeer, Jennifer Pereira, Susan Quach, Sherman Quan, Beate Sander, Chris Sikora, Anne-Luise Winter.

## Pre-publication history

The pre-publication history for this paper can be accessed here:

http://www.biomedcentral.com/1472-6947/12/145/prepub
